# Risk of Injury to Retroperitoneal Structures in Prone and Lateral Decubitus Transpsoas Approaches to Lumbar Interbody Fusion: A Pilot Cadaveric Anatomical Study

**DOI:** 10.7759/cureus.41733

**Published:** 2023-07-11

**Authors:** Luiz Pimenta, Samuel A Joseph, Jeffrey A Moore, Jack Miles, Jorge E Alvernia, Kelli M Howell

**Affiliations:** 1 Spine Surgery, Instituto de Patalogia da Coluna, São Paulo, BRA; 2 Spine Surgery, Joseph Spine Institute, Tampa, USA; 3 Spine Surgery, SonoSpine, LLC, Oklahoma City, USA; 4 Clinical and Scientific Affairs, ATEC Spine, Carlsbad, USA; 5 Spine Surgery, Brain and Spine Associates, LLC, Monroe, USA

**Keywords:** potential space, kidney injury, bowel injury, peritoneum, complications, single-position surgery, prone lateral, ptp, ltp, llif

## Abstract

Introduction

The retroperitoneal approach for lateral lumbar interbody fusion (LLIF) originally described an initial posterolateral fascial incision enabling finger dissection from behind the peritoneum and guidance of instruments through a second direct-lateral fascial incision. It has since become common for single direct-lateral incisional access to the retroperitoneum. This study attempted to quantify the distance of the peritoneum from posterior landmarks in the space, assess the risk of peritoneal violation in each access trajectory (i.e., posterolateral versus direct lateral retroperitoneal dissection), and determine whether there are differences based on patient position (prone versus lateral decubitus).

Methods

In three prone cadaveric torsos, Steinman pins were percutaneously placed mid-disc at each level L2-5 bilaterally (for a total of 18 prone approaches). Open dissections exposed the retroperitoneum including the quadratus lumborum and psoas muscles, maintaining the natural reflection of the peritoneum. Visual assessment qualified whether any pin violated any retroperitoneal structure. Distance from the anterior border of the quadratus lumborum to the posterior-most reflection of the peritoneum was measured. For comparison, three additional torsos were positioned in lateral decubitus, and the above steps were repeated, only unilaterally (for a total of nine lateral decubitus approaches).

Results

In prone, no pin violated the peritoneum; three (3/18 total approaches) violated the kidney, all at L2-3 (3/6 approaches at L2-3). In lateral decubitus, all three L2-3 pins violated the kidney (3/3 approaches at L2-3); five of the six remaining pins from L3-5 violated the peritoneum (totaling eight violations in the nine total approaches). The incidence of any violation was significantly greater in lateral decubitus vs. prone (8/9 vs. 3/18, p=0.0006). The structure at risk (kidney vs. peritoneum) was significantly associated with disc level (p=0.0041): all kidney violations occurred at L2-3 and all peritoneal violations occurred at L3-4 or L4-5. Distance from the quadratus lumborum to the posterior-most reflection of the peritoneum averaged 8.7 cm (range: 6-10) in prone, and 2.9 cm (range: 2.5-3.2) in lateral decubitus (p=0.0129).

Conclusion

A cadaveric study of retroperitoneal anatomy demonstrates that there is an increased distance from the quadratus lumborum to the peritoneum in prone versus lateral decubitus and that the trajectory of approach to the lumbar discs risks violation of the peritoneum more frequently when accessing directly laterally versus posterolaterally. In either approach, care should be taken to identify and release the peritoneal reflection to create a safe passage to the lumbar discs.

## Introduction

Retroperitoneal access for surgery to the anterior column of the lumbar spine has become common since first described for stabilization of Pott’s disease nearly a century ago [1,2] and has advanced through the introduction of laparoscopic techniques [3] and subsequent minimally invasive muscle-splitting approaches [4]. Even in the earliest descriptions, a traverse of the extraperitoneal space was advocated over transperitoneal exposures to avoid “contamination of the peritoneum” [1].

Modern anterolateral access techniques, such as lateral lumbar interbody fusion (LLIF) and oblique lateral interbody fusion (OLIF), have gained popularity for the reconstructive advantages of anterior column correction coupled with minimally invasive benefits [5]. LLIF, in particular, avoids the anterior vasculature altogether by accessing the disc space through the psoas muscle.

The retroperitoneal access technique for LLIF was originally described by Pimenta as through the use of two separate fascial incisions: the first, posterolateral access to digitally palpate and expand the retroperitoneal space; and the second, direct lateral access through which the initial dilating instrument is guided by the finger already in the space to mitigate the risk of injury to the peritoneum [6]. Over the years since this was first described, modification of the approach included the use of single direct-lateral access into the retroperitoneal space, using careful dissection and visualization to avoid peritoneal injury [7].

The risk during LLIF surgery of peritoneal injury, or to the contents within it, is reportedly low, at well less than 1% [8]; however, complications do occur [8-10]. Moreover, more recent descriptions of LLIF surgery being performed with the patient in the prone position [11] have renewed concerns regarding the potential risk to retroperitoneal structures, especially in comparison across the techniques.

The current study was undertaken to investigate the anatomical position of the posterior reflection of the peritoneum as it relates to direct-lateral access to the lumbar spine for LLIF surgery and to attempt to identify methods to mitigate the risk of a peritoneal violation through alternative anatomical landmarks. Differences in anatomical positions and spaces were evaluated between lateral and prone decubitus.

## Materials and methods

Three fresh-frozen (not chemically preserved), unoperated, adult cadaveric torsos with thighs were procured from an accredited and licensed tissue bank. These specimens were fully thawed and positioned prone on a Jackson-style surgical frame using a bolstering system developed for prone lateral surgery (ATEC Spine, Carlsbad, CA) that allows for the abdominal contents to fall anteriorly through the frame while securing the thorax and pelvis. The bolsters allow for controlled coronal bending between the thoracic and pelvic positioners as needed for lateral access to the lumbar spine; however, in this case, the torsos were positioned coronally neutral, and with thighs in neutral alignment (i.e., neither flexed nor extended).

**Figure 1:**
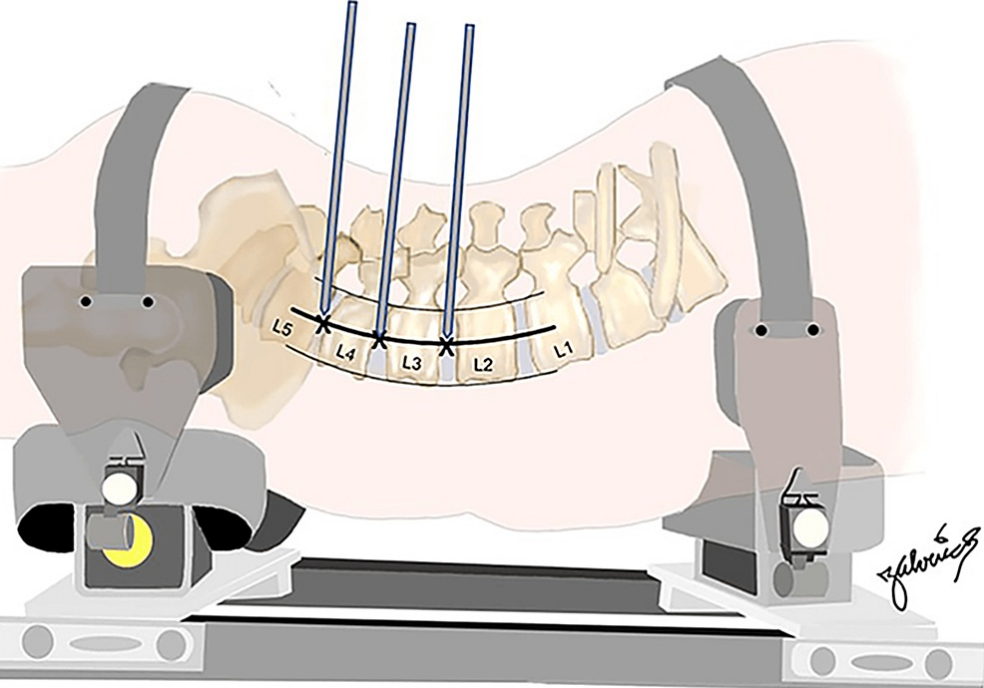
Illustrative representation of lateral insertion of Steinman pins targeting the center of the L2-3, L3-4, and L4-5 disc spaces. Courtesy: Dr. Jorge. E. Alvernia

Prior to dissection, the lumbar disc spaces from L2-3 to L4-5 were identified fluoroscopically and Steinman pins were placed into the disc spaces percutaneously, attempting an orthogonal approach to the center of each disc space (Figure 1) - simulating a worst-case-scenario blind approach without the typical dissection and visual and tactile expansion/development of the retroperitoneal space and confirmation of avoidance of the peritoneum or its contents during a LLIF approach. This was done bilaterally such that six approaches were made in each of the three cadavers, for a total of 18 approaches.

**Figure 2:**
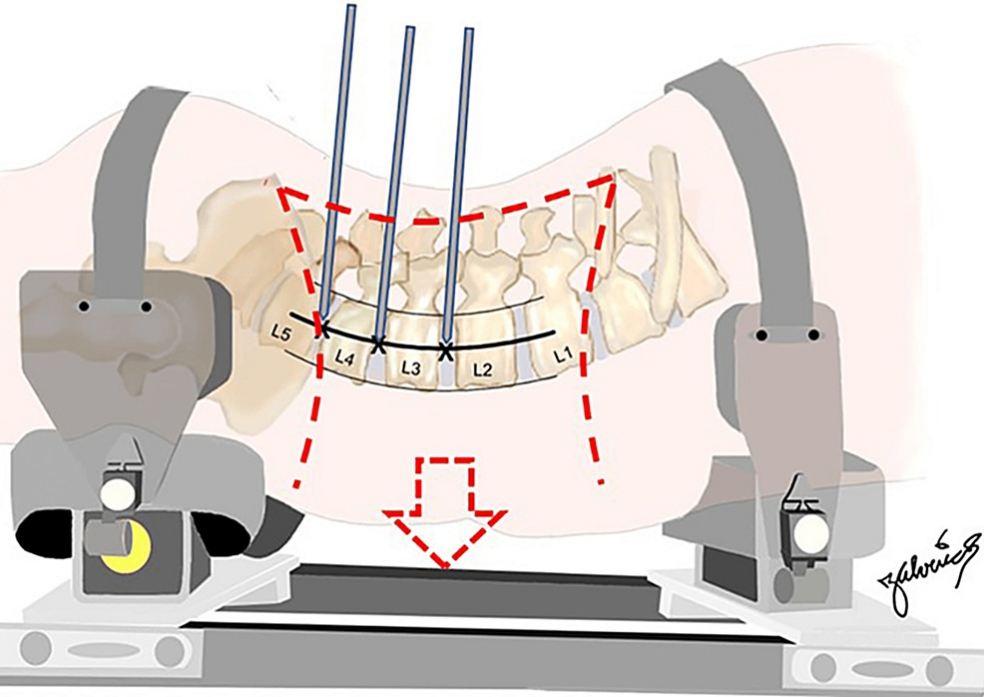
Illustration of dissection perimeter to expose the retroperitoneum. Courtesy: Dr. Jorge. E. Alvernia

**Figure 3:**
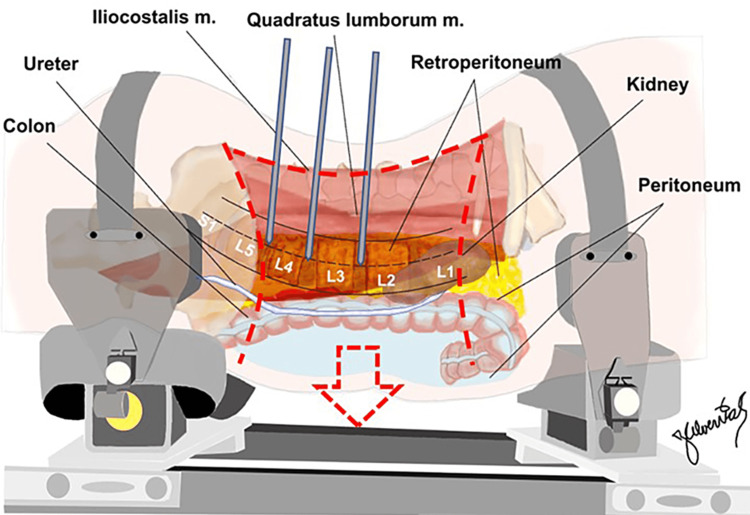
Illustrative representation of the retroperitoneal anatomy exposed via the flap incision. Courtesy: Dr. Jorge. E. Alvernia

With the pins in place, open dissections were performed by an experienced spine surgeon, exposing along the transverse lines of the 12th rib and the iliac crest, and extending posteriorly to the border of the erector spinae muscles (Figure 2), exposing the retroperitoneum including the quadratus lumborum and psoas muscles (Figure 3).

**Figure 4:**
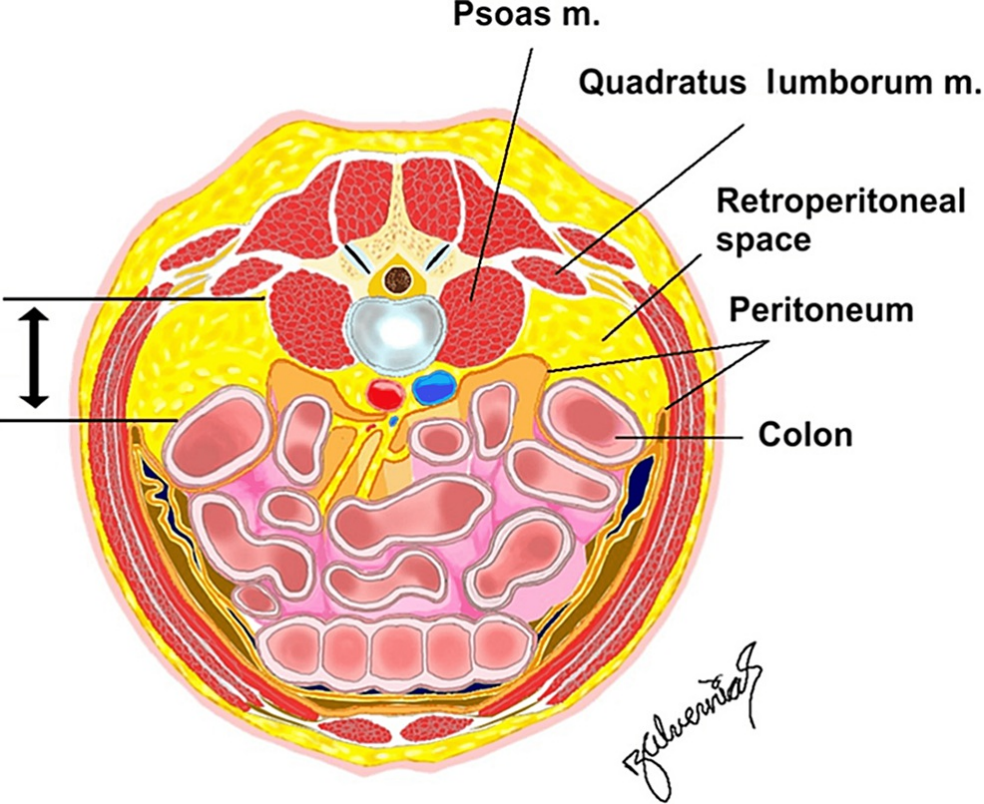
Illustration in the axial plane showing the distance measurement from the quadratus lumborum to the posterior-most reflection of the peritoneum. Courtesy: Dr. Jorge. E. Alvernia

Visual assessment qualified each Steinman pin placement as free or not free of retroperitoneal anatomy. The distance from the anterior border of the quadratus lumborum to the posterior-most reflection of the peritoneum was measured (Figure 4).

For comparison, three additional torsos were positioned in lateral decubitus consistent with traditionally described LLIF technique of taping over a break in a bending table [6]. The above steps were again taken, however only unilaterally. Statistical analysis included comparison of means for continuous variables (e.g., distance measurements) and chi-square test for association among categorical variables.

## Results

The findings are shown in Table 1.

**Table 1:** Incidence of violation of any retroperitoneal structure, by intervertebral disc level and by patient decubitus QL=quadratus lumborum muscle; P=psoas muscle

	Lateral Decubitus		Prone	
	# Approaches	# Violations	# Approaches	# Violations
L2-3	3	3 (kidney)	6	3 (kidney)
L3-4	3	3 (peritoneum)	6	0
L4-5	3	2 (peritoneum)	6	0
Avg QL-P Distance*	2.9 cm		8.7 cm	

Visual assessments

In the prone position, none of the blindly placed Steinman pins violated the peritoneum (0/18). However, three (3/18) violated the kidney - all occurring at the L2-3 level. In the lateral decubitus position, all three L2-3 approaches also resulted in kidney violation; additionally, approaches at five of the six remaining levels from L3-5 resulted in the violation of the peritoneum (bordering on the bowel). The incidence of there being any violation of a retroperitoneal structure was significantly greater in lateral decubitus versus prone (8/9 vs. 3/18, p=0.0006). The structure at risk (kidney versus peritoneum) is significantly associated with level (p=0.0041), where all kidney violations occurred at the L2-3 level, and all peritoneal violations occurred at L3-4 or L4-5.

Distance measurements

**Figure 5:**
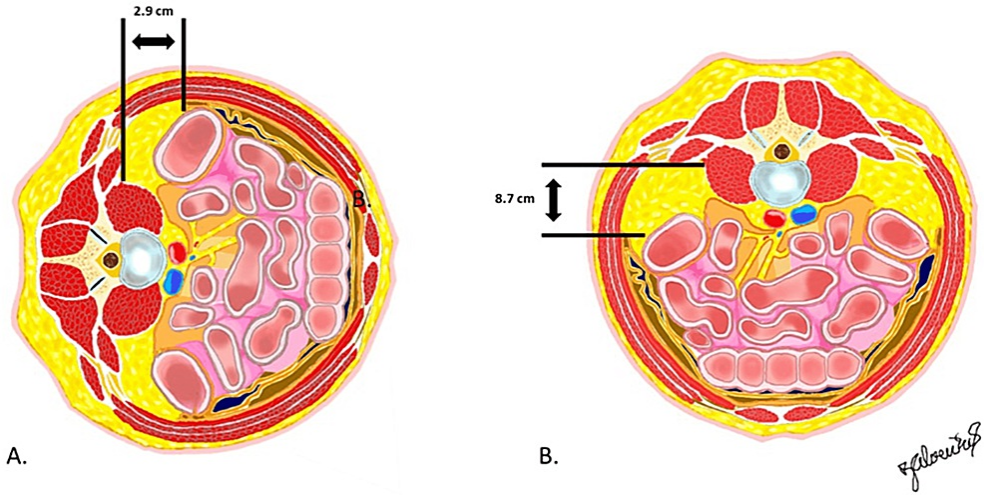
Illustrations in the axial plane showing the distance measurement from the quadratus lumborum to the posterior-most reflection of the peritoneum found in lateral decubitus (A) and in prone decubitus (B). Courtesy: Dr. Jorge. E. Alvernia

The distance from the quadratus lumborum to the posterior-most reflection of the peritoneum was on average 2.9 cm (range 2.5-3.2 cm) in lateral decubitus, and 8.7 cm (range 6-10 cm) in prone (p=0.0129) (Figures 5A, 5B).

## Discussion

Despite the common approach corridor, there has been surprisingly little published on the anatomy of the retroperitoneal space. Most anatomical studies relating to LLIF surgery have been with respect to neurological and/or vascular risks, most in an attempt to define a “safe zone” for access into the disc space [12-14].

Few reports exist related to other retroperitoneal anatomical structures. Iwanaga and Tubbs summarized a series of cadaveric studies investigating the anatomical locations of the kidney, ureter, and colon relative to the lateral disc space [15]. In each study, they placed metal wires into the center of the lumbar disc spaces of a variable number of cadavers and measured distances to anatomical structures. They identified that the kidneys and ureter are closest to the lateral aspect of the disc at the most cranial levels and increasingly distant so as to be anatomically irrelevant at the L4-5 disc space [16,17]. Separately, they determined a similar scenario with distance from the colon, except that the L1-2 disc level was above the colon on both sides, but the greatest risk of injury to the colon was determined to be at the L2-3 or L3-4 levels [18].

The current study similarly used metal pins in the disc space as a representation of LLIF access. In Iwanaga et al.'s studies, the pins were placed after the dissection of the retroperitoneal space. In the current study, they were placed before, in an attempt to identify the risk of a blind direct-lateral approach by observing direct impingement of anatomical structures. Upon dissection, it was observed that six of nine approaches to the L2-3 level violated the kidney (three of three in lateral decubitus and three of six in prone), and five of 27 total approaches violated the peritoneum (at both the L3-4 and L4-5 levels, all in the lateral decubitus position).

It was a limitation of this study that an equal number of approaches in each decubitus was not performed (due only to the requirement to reposition the lateral decubitus specimens, which was not an issue for the prone specimens). Despite the difference in cohort size, it was an advantage of the current study to include the comparative assessment based on patient decubitus. That comparison found that the incidence of any anatomical structure violation from a blind direct-lateral approach was statistically greater in lateral decubitus than in prone. Prone positioning on an open-frame table is intended to allow the abdominal contents to fall anteriorly away from the spine with gravity, which seems to have allowed anterior migration of both the kidney and the peritoneum in these specimens more than did lateral decubitus. To the authors’ knowledge, there are few studies that have identified variances in anatomical structure location based on patient decubitus.

Deukmedjian et al. showed through magnetic resonance images (MRI) that the kidneys and vasculature do move between supine and lateral decubitus, but the study did not include prone decubitus [19]. Ghazi et al. cautioned that the location of the lateral peritoneal reflection should be critically considered when attempting a retroperitoneal approach, summarizing the findings of Chiu et al. and Capelouto et al. that its position varies with patient decubitus [20]. Specifically, both Chiu and Capelouto demonstrated that the movement from supine to lateral decubitus increased the distance between the quadratus lumborum and the colon [21] and the peritoneal reflection [22], increasing the anteroposterior dimension of the potential retroperitoneal space twofold, presumably due, according to Ghazi, to “gravity-induced downward movement of the ipsilateral colon causing anterior displacement of the mesocolon and thus its peritoneal reflection” [20].

More recently, Dodo et al. reported on the anatomical positional changes seen between supine preoperative and prone intraoperative computed tomography (CT) scans, finding that retroperitoneal organs shifted ventrally with prone positioning [23]. They cautioned, however, that the shift did not eliminate all risks of organ injury as the location of some organs was still in the path of a theoretical direct lateral interbody cage insertion. It is worth noting that Dodo’s study was a retrospective review of imaging performed for patients undergoing surgical procedures other than prone LLIF surgery. The intraoperative positioning did not employ the specific bolsters or attention to allowing the abdomen to freely hang through a Jackson frame-style surgical table as would be done for the prone transpsoas procedure (PTP) [11]. Nor did the surgical exposures include retroperitoneal access which might encourage gravitational pull of the abdominal contents anteriorly. The results of Dodo et al.'s study [23] underscore the findings and message from the current study, which is that the retroperitoneal space need to be intentionally developed for safe surgical access to the lumbar spine.

**Figure 6:**
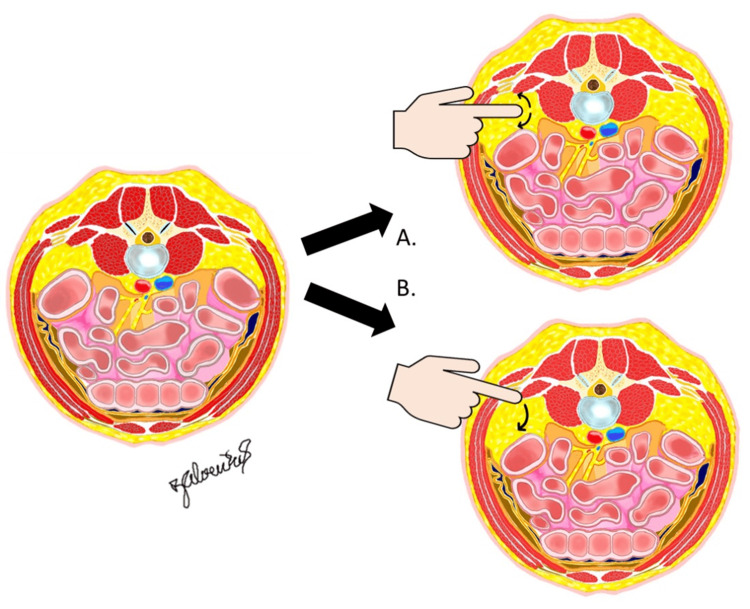
Illustrations in the axial plane showing how the retroperitoneal space can be developed through finger palpation and sweeping movements either through a direct lateral approach in an attempt to reach the psoas muscle directly (A) or through an initial posterolateral trajectory to first reach the quadratus lumborum as a safe landmark and then sweeping anteriorly (B). Courtesy: Dr. Jorge. E. Alvernia

The retroperitoneal space is that space bordered by the posterior parietal peritoneum anteriorly and the transversalis fascia anterior to the quadratus lumborum muscle posteriorly and extending to the liver cranially and the pelvic brim caudally [24]. It must be remembered that the retroperitoneal space is not a natural cavity but rather a “potential space” [25], requiring the release and mobilization of relative tissue planes to define and develop the “space.” The peritoneum itself reflects along the abdominal wall from anterior to posterior, and so could potentially be found directly underlying a direct-lateral access to the lumbar spine (as was found in five of 27 blind lateral approaches to the lumbar disc spaces). Of course, surgeons do not typically blindly access the retroperitoneal space, but rather do or should work to develop the potential space to release any adherent tissues and enable gravitational movements of the abdominal structures (Figure 6A, 6B).

The current study’s authors have been advocates of the two-fascial-incision and finger dissection technique since the introduction of the LLIF procedure in the early 2000s [6]. It is intuitively anatomically safer (specific to the peritoneum), although does add time and steps to the procedure, and many surgeons have found success with the direct-lateral single-incision dissection approach. As mentioned, the reported rate of complication to non-neurological retroperitoneal structures is low. Bowel injuries had been reported only as isolated case reports [9] or within small series of complex surgery [10]. Uribe et al. employed the methodology of a societal survey to try to account for the relative incidence of significant complications including visceral and vascular injury [8]. In that survey of over 13,000 retroperitoneal LLIF surgeries performed, the incidence of bowel injury was 0.08% [8]. The authors noted that the majority (70%) of those injuries occurred when the procedure was performed using a single-incision approach, although they could not attribute that to the cause.

The OLIF approach has been described as an alternative to direct-lateral LLIF to avoid crossing the psoas muscle and thereby avoiding the lumbar plexus. However, the more anterior access approach has resulted in a higher incidence of vascular and visceral injuries, including ureteral injury and peritoneal laceration [26,27].

It seems that surgeons have come to accommodate their own preferences for risk avoidance based on their training and skills for what works in their own hands. The more recent introduction of a technique to perform LLIF surgery with a patient in prone decubitus [11], however, has raised new questions about the potential risks to these anatomical structures, presumably based on concern for posterior compression rather than anterior migration of the peritoneum. The current study dispels that concern, showing that the peritoneum was free from violation in all 18 prone approaches, yet was violated in five of nine lateral decubitus approaches. This finding was underscored by the measured distance of the posterior reflection of the peritoneum from the quadratus lumbar muscle: 2.9 cm in lateral decubitus and 8.7 cm in prone.

This distance from the quadratus lumborum to the peritoneum demonstrates the relative anterior expansion of the retroperitoneal space in prone decubitus, but it should not suggest that blind direct-lateral access is safer. It has been the authors’ experience that the distance from the skin to the spine in a direct lateral trajectory is sometimes greater when the patient is prone versus in lateral decubitus, such as in larger habitus patients where, without proper soft tissue management, there can be a flattening out of the abdominal wall/pannus. When this happens, it can be difficult to reach with a finger into the retroperitoneal space and palpate the psoas muscle (as is typically done in LLIF to ensure that the path is clear of important tissues). For this reason, the second, posterolateral, fascial incision becomes even more relevant than in traditional lateral decubitus LLIF. Approaching the retroperitoneal space at the quadratus lumborum allows the palpation forward to release the peritoneal posterior reflection and to guide the initial LLIF dilator to the surface of the psoas muscle, which can be more readily reached from this trajectory.

## Conclusions

The current study is the first to measure the location of the peritoneum, and its implications for safe retroperitoneal LLIF access, based on decubitus. The risk of visceral violation during retroperitoneal access for LLIF is not increased in prone versus lateral decubitus. The prone position appears to allow for increased anterior migration of the peritoneal reflection compared to lateral decubitus. Care should be taken in either position to avoid the kidney when at the L2-3 level. The risk of visceral violation may be reduced if the approach trajectory starts posteriorly (at the quadratus lumborum) compared to directly lateral to the disc, regardless of surgical position. Note that in either position, careful digital palpation and release of tissues are important steps to the creation and development of the retroperitoneal potential space to become a safe and functional working space.
